# Notes on Female Circumcision as Practised by the Ameru

**Published:** 1932

**Authors:** H. W. Brassington

**Affiliations:** Mana, Kenya Colony


### NOTES ON FEMALE CIRCUMCISION AS PRACTISED BY THE AMERU.

BY

H. W. Brassington, M.B., Ch.B.,

*Mana, Kenya Colony.*

---

The Ameru are a tribe inhabiting the northern part of the Kikuyu Province of Kenya Colony. The area of this part of Kikuyu, called Meru, is 3,500 square miles, and has about 110,000 inhabitants. The southern boundary of this district is within about thirty miles north of the Equator, and the northern boundary is about eighty miles north. The Ameru are a very primitive people, and are making a last effort to conserve ancient customs, be they good, bad or indifferent.

At the present moment they are making a determined effort to conserve the custom of female circumcision. This custom is universal amongst certain of the Bantu tribes, to which the Ameru belong, yet no basis for the custom has been found. The Church of Scotland Mission at Kikuyu suggest that the custom is comparable to that of the Nubians of sewing up the vulva when a woman becomes pregnant or her husband leaves the home to seek work at some distant spot, and ensures the fidelity of the woman. A superficial examination of this theory is enough to kill it, because until recent years there was no marital infidelity on the part of the women. One reason for the retention of the rite is the belief that it increases fertility on the part of the woman. The main point, with which these people are concerned now, is that it is an essential to the entrance of women into full tribal membership. Should a child be born to an uncircumcised woman, neither she nor the child can claim a single tribal privilege, and should any children be born to this woman after circumcision and marriage they will die on account of the child born prior to the rite of circumcision. That this can happen is proved by the fact that there is a man in Government employ who was born while his mother was still uncircumcised. After circumcision she was married and gave birth to four more children, all of whom are dead from this form of suggestion. The man is known to myself, so that I can vouch for the truthfulness of the history.

The fact that no child of an uncircumcised woman can enter into tribal rights has led to the almost universal practice of abortion. This is due to the fact that when a man's daughter reaches a certain age he announces the fact, and she is then at liberty to be taken by a warrior as his mistress without any obligation to marriage. Should she become pregnant by this lover, he must give the father a goat or sheep, but the seriousness of this pregnancy arises from the fact that unless the woman is circumcised the child will be an outcast. To prevent this the woman takes an abortifacient, or the warrior may exert pressure over the womb with a view to causing the expulsion of the fœtus. Until recently it was not unknown for the man to give the woman a sharp blow over the womb, and this often led to her death.

When a girl is sought in marriage the man has to pay the father of the girl a certain number of cattle, sheep or goats, and at some time prior to the completion of the purchase the girl has to be circumcised. A day for the ceremony is fixed, and the woman who will perform the operation is given a price for it. In this district the circumciser receives a goat's skin worth about two shillings, some food to the value of about sixpence and a few beads bought at the nearest Indian shop. About forty-eight hours before she is due to be circumcised the girl rubs herself liberally with castor oil until her whole body appears to have been black-leaded. She then adorns herself with a striking headdress, brass wire armlets and leglets, and on her knees she fixes some metal containing iron balls to make a noise as she walks. In her hand she carries the end of a cow's tail, and round her middle she has a narrow fringe of leather. She then sets out on parade, and at intervals she stops to dance and sing. The dancing consists of swaying on her toes and pushing the abdomen backwards and forwards. This goes on until just before the ceremony, by which time she is really excited and tired. She now receives the second bath of her life, the first being four days after birth. After this ceremonial washing at a convenient stream she is led by the older women, who are adorned with shields and swords or sticks, to the place of the ceremony, where she will be met by a large crowd of females of all ages. During all this there is continuous dancing and singing. At or near 4 p.m. the girl is stripped naked except for a string of beads round her middle, and after a few minor rites takes her place within a small circle of women, one of whom will support her back as she sits on the ground with legs abducted. The operator, a dirty woman with a dirtier knife, now approaches and makes a cut which removes the clitoris and the whole of the labia minora. As the cut is being made the entire host of females utters a yell to drown the cry of the one who is being cut. After this the girl is draped in a dirty black cowhide and escorted to a dark and dirty hut, still bleeding profusely, and here she stays for about a month with the one who supported her during the operation. During her stay in this hut she receives instruction in the tribal rites and laws and also marriage obligations. After the wound has healed she can then be taken by her husband.

Sometimes it happens that the operator is over zealous and then she may cut into the vault of the vagina. In illustration of this I may mention the case of a woman of between 25 and 30 years of age who came to me on account of constant dribbling of the urine from the vagina. When I examined her I found that it was with great difficulty that one finger could be passed into the vagina. The reason for this was a circular band of dense fibrous tissue about one inch deep where the labia minora should have been. In addition I found that there was a similar band of hard fibrous tissue in the vault, and this surrounded a hole leading into the bladder. This woman thought that a dose of medicine would cure her trouble, and she refused any radical treatment. She was, I found out later, a prostitute, and whenever she became pregnant resorted to abortifacients. I saw another woman about the same age just after labour had terminated and found that she had a fairly large perineal tear. On examining her more carefully I found that in place of the labia minora there was a very well marked and sharp band of fibrous tissue. She refused to have the tear sutured, and when last I heard of her the wound had healed satisfactorily. In the case of breach presentation labour is long and tedious, even when the fibrous band is thin. I have had two such cases ; fortunately, each labour terminated with a living child, but the second one was " touch and go " after a very distressing labour.

I have made no comments on this practice, but have tried to give some sort of picture which, I hope, is clear enough to convey to any who may read these simple notes some of the burden of the women of this tribe. Strange to relate, it is they and not the men who are the strongest protagonists of this horrible and mutilating custom. It remains only to be said that at the beginning of this year the local native council decreed that only certificated circumcisers should perform the operation, and that the operation be limited to clitoridectomy.

